# Supplementary material for: Absence of Figla-like Gene Is Concordant with Femaleness in Cichlids Harboring the LG1 Sex-Determination System
Source: Int J Mol Sci. 2022 Jul 11;23(14):7636. doi: 10.3390/ijms23147636 (PMC9316214; doi:10.3390/ijms23147636)
Supplement: Supplementary file 1 [file ijms-23-07636-s001.zip › Figures S1-S4.pdf]

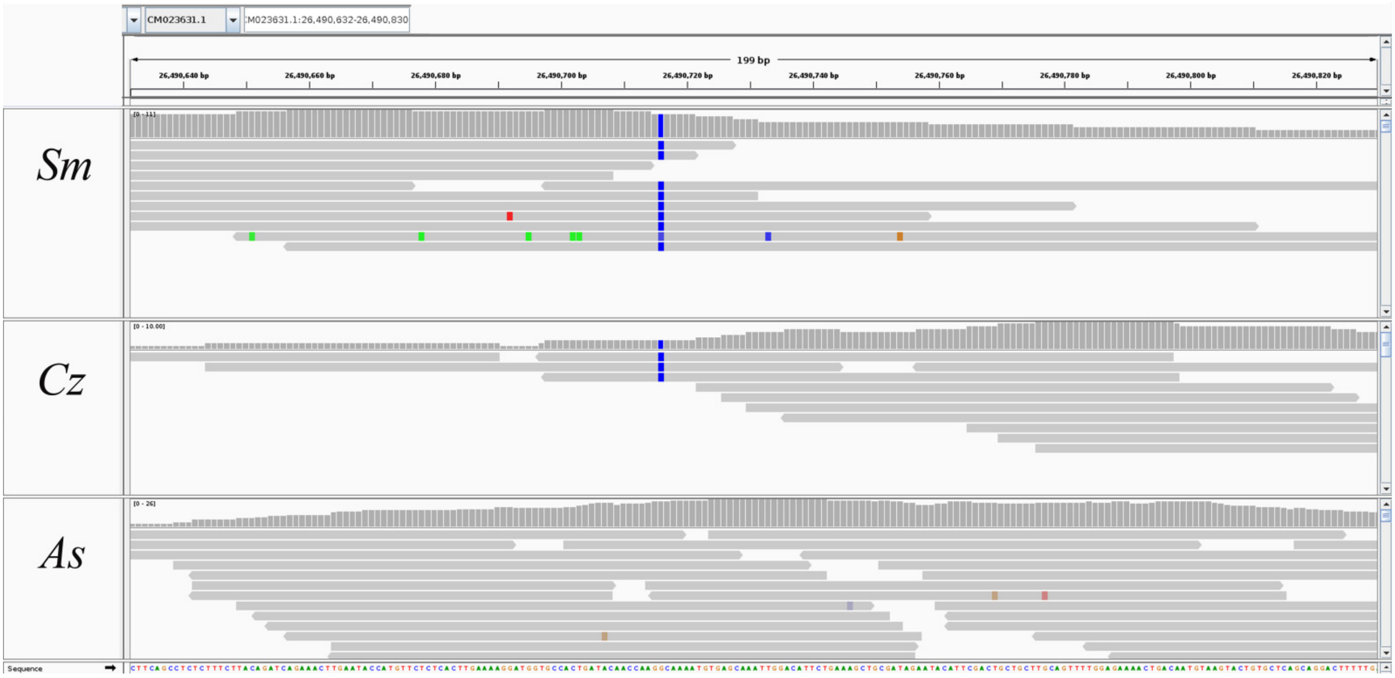

Figure S1. Integrative-Genomics-Viewer (IGV) output exemplified for alignments of *figla-like* exon 2 of sequence reads from males of *Sarotherodon melanotheron* (*Sm*), *Coptodon zillii* (*Cz*) and *Amherst strain* (*As*). Reference sequence and positions are indicated above (Nucleotide accession # CM023631.1, top left), whereas the consensus sequence is shown below. For each alignment, the aligned reads and their orientations are delineated as gray arrows, below the coverage histogram. Nucleotide differences from the consensus are indicated by colored boxes: green, orange, red and blue for adenine, guanine, thymine and cytosine, respectively. Intensity of colors indicates the base quality of the recorded SNP. At position 26490716, in the coverage histograms, the blue bars denote the variation encoding R30S (Table 1, Figure 2a).

```
G2        ---------------------------------------------------------------------
G1        ---------------------------------------------------------------------
G3        ---------------------------------------------------------------------
Ot        ---------------------------------------------------------------------
Pm        ---------------------------------------------------------------------
figla Oa  ----------------MKVPEEESMSDILKRLTGESALPVYCNIEKLKRGRDNLYFVAEEFSETVKKRE
figla Dre MSCEMTGKKFRAIVGLMKVPERELMGDVLLRESAEPSLPVHVNIGKFVRLDDGQYIDTEDRDKAVKRRQ
figla Mm  ----------------------------------MGPLPRLAAICRLKRLPSGGYSTTDDLHLVLERRR
          1........10........20........30........40........50........60........

G2        MNQNENNGTIRNLNTMFSHLKRMVPLIQPSQNVSKLDILKAAIEYIRLLLAVLEKT-DNDEGSVTDFLK
G1        MNQNENNGTIRNLNTMFSHLKRMVPLIQPSQNVSKLDILKAAIEYIRLLLAVLEKT-DNDEGSVTDFLK
G3        MNQNENNGTIRNLNTMFSHLKRMVPLIQPRQNVSKLDILKAAIEYIRLLLAVLEKT-DNDEGSVTDFLK
Ot        MNQNENNGTIRNLNTMFSHLKRMVPLIQPRQNVSKLDILKAAIEYIRLLLAVLEKT-DNDEGSVTDFLK
Pm        MNQDENNGMIRNMNTMFSHLKRMVPLIQPSQNVSKLDILKAAIEYIRLLLAVLEKT-DDDEGSVTDFLK
figla Oa  LVNAKERMRIRNLNTMFSRLKRMVPLMRPDRKPSKVDTLKAATEYIRLLLAVLQET-DSDDGSGTDFLK
figla Dre LANAKERLRVRSLNSMFSYLRRIVPVMPRDRKPSKVDMLKAATEYIRLLSAVLNHTSDKGNENANVFLE
figla Mm  VANAKERERIKNLNRGFAKLKALVPFLPQSRKPSKVDILKGATEYIQILGCVLEEAKVSEKQSPEEQTH
          70........80........90........100.......110.......120.......130.....

G2        NAIIYSQAEGLGSDLWQVNDVDDDD-KVAVSP-------------------------------------
G1        NTIIYSQAEGLGSDLWQVNDVDDDD-KVAVSP-------------------------------------
G3        NAIIYSQAEGLGSDLWQVNDVDDDD-KVAVSP-------------------------------------
Ot        NAIIYSQAEGLGSDLWQVNDVDDDDDKVAVSP-------------------------------------
Pm        NAIIYSQAEGLGSDLWQVNDVDDD---VAVSP-------------------------------------
figla Oa  NAITYSQTEGLGSDLWRVDDWNREETLMELLSMSEEQIDDGFTLPPEPVTDNGEMTRLMLQHCVMPTYQ
figla Dre TGINYPSINPDCSDLWNMEDALDSMSSPFLSETVSLTAP---LVTMTSGADEDLDLNNMTVQCVVPMYI
figla Mm  SGRPSDPHVSSTRELLGNATQPTSCASGLKKE----------EEGPWAYAGHSEPLYSYHQSTVPETRS
          .140.......150.......160.......170.......180.......190.......200.....

G2        ----------------
G1        ----------------
G3        ----------------
Ot        ----------------
Pm        ----------------
figla Oa  FIIQVAPDQPATSQPC
figla Dre VQVGSDQTMVPSSLPS
figla Mm  YFTH------------
          ..210.......220.
```

Figure S2. An alignment of the predicted proteins encoded by *figla-like* and *figla* proteins. The alignment includes five shorter polypeptides, which are of *figla-like* protein groups (G1-4). Protein group G1 (blue) consists of the identical polypeptides of *C. zillii* (*Cz*) (Nucleotide accession number: OW742498) and *S. galilaeus* (*Sg*) (Nucleotide accession number: OW742804). Protein group G2 (red) consists of the identical polypeptides of *O. urolepis hornorum* (*Oh*) (Nucleotide accession number: OW740593) and *O. mossambicus* (*OmI*) (Nucleotide accession number: OW739941). Protein group G3 (green) consists of the identical polypeptides of *Chitralada strain* (*Cs*) (Nucleotide accession number: OW739608), *O. aureus* genomic build (*LOC116310109*), *O. aureus* from *Ein-Feshka* (*Oa Ein-Feshka*) (Nucleotide accession number: OW770257) and *Amherst strain* (*As*) (Nucleotide accession number: OX031319). *O. tanganicae* (*Ot*, green) (Nucleotide accession number: OW739839) is shown out of G3 as its sequence differs by an additional D residue. *P. mariae* (*Pm*, purple) (Nucleotide accession number: OW742294) is the only member in G4. The alignment also includes three longer *figla* polypeptides for *Oa* (Protein accession number: XP_039476449.1), *Danio rerio* (*Dre*) (Protein accession number: NP_944601.2) and *Mus musculus* (*Mm*) (Protein accession number: NP_036143.1). Dashes indicate gaps introduced by the alignment program. Identical amino-acid residues in at least four of eight sequences are indicated by a black background. White boxes indicate non-conservative amino-acid changes between the proteins, whereas gray boxes indicate conservative changes.

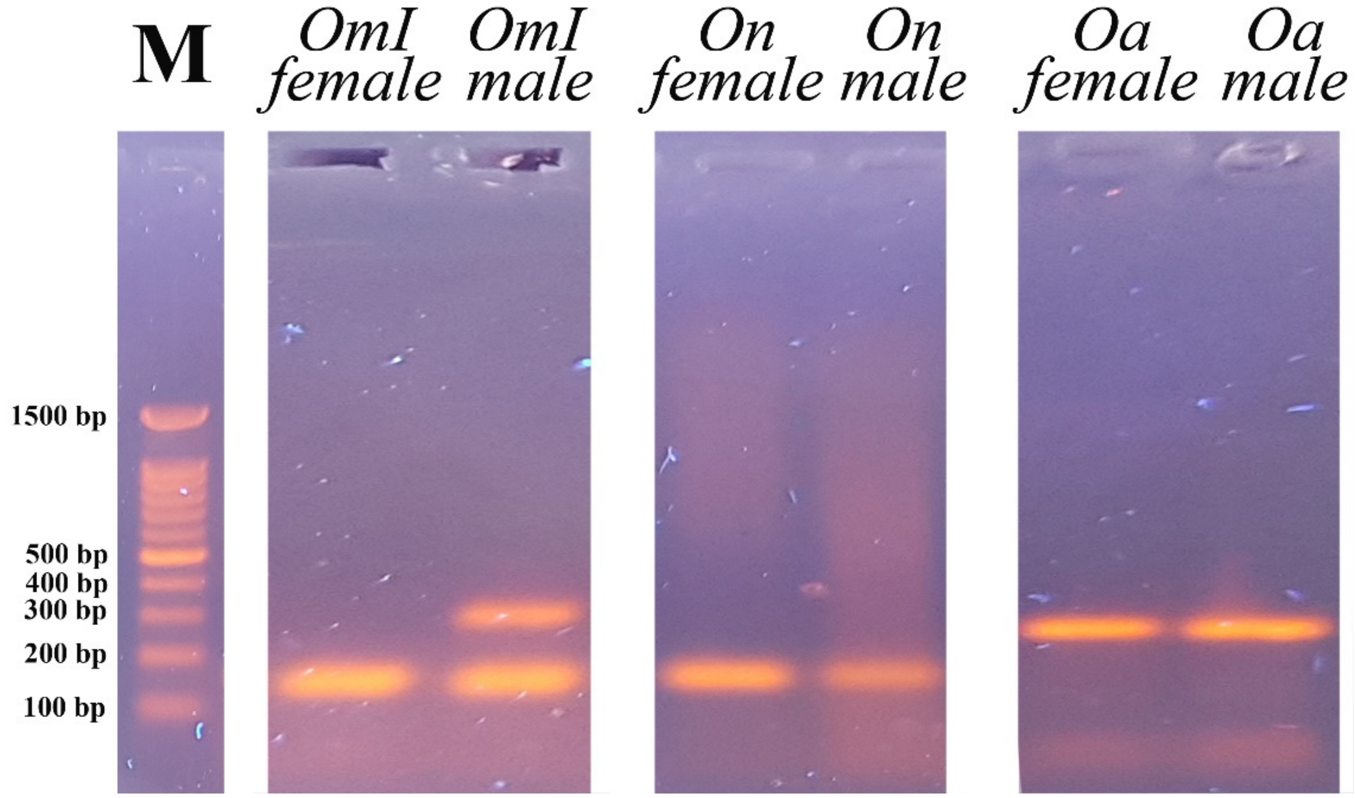

Figure S3. Diagnostic duplex marker for the detection of LG1y and LG1x in purebred and hybrid cichlids. On the left size a genomic molecular ladder (M, 100bp – 1.5Kbp). Samples: *O .aureus* (*Oa*), *O. niloticus* (*On*) and *O. mossambicus* (*Om*). The marker worked efficiently also in *S. galilaeus*. Primer sequences are given in Table 3.

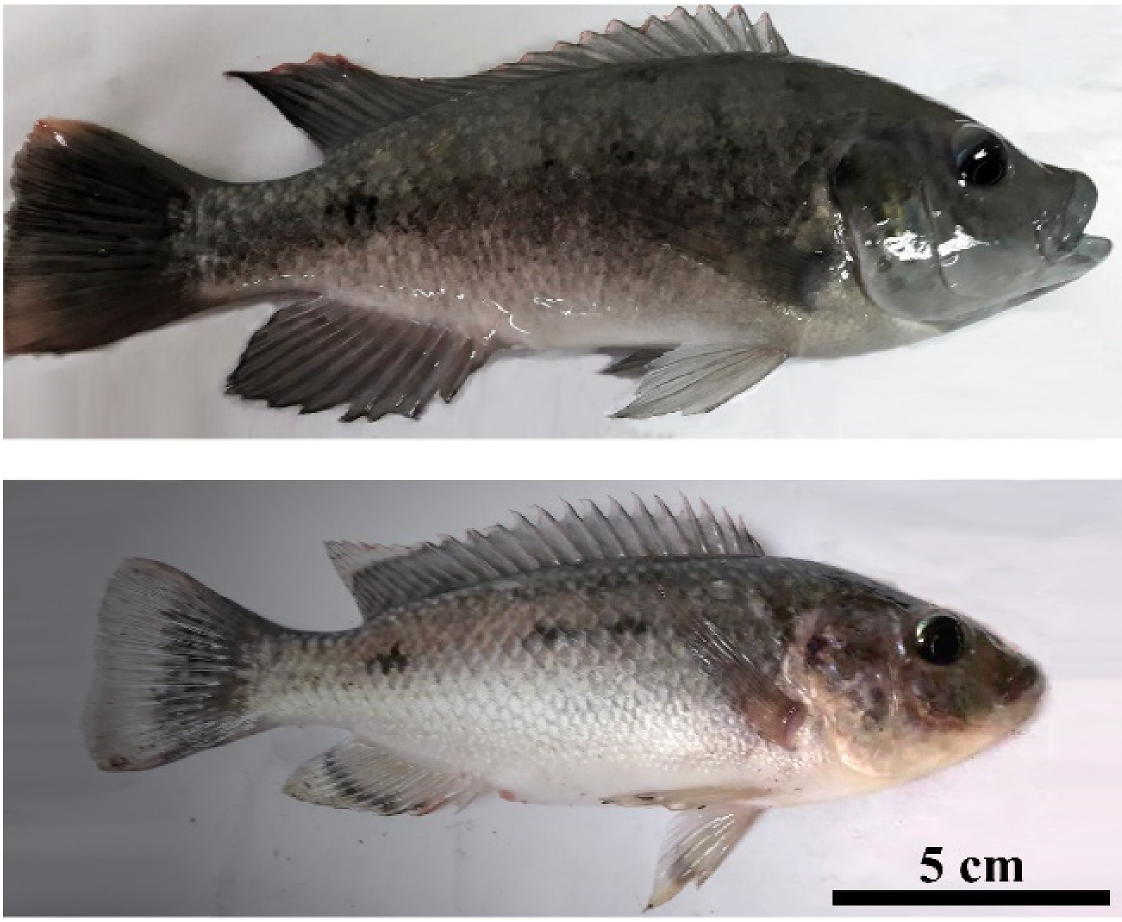

Figure S4. *Oreochromis mossambicus* (Type 1) male (top) and female (bottom) grown in Volcani Institute of Agriculture (ARO). This *Oreochromis mossambicus* is referred to as *OmI* in the study.
